# Supplementary material for: Effect of Early Supraglottic Airway Device Insertion on Chest Compression Fraction during Simulated Out-of-Hospital Cardiac Arrest: Randomised Controlled Trial
Source: J Clin Med. 2021 Dec 31;11(1):217. doi: 10.3390/jcm11010217 (PMC8745715; doi:10.3390/jcm11010217)
Supplement: Supplementary file 1 [file jcm-11-00217-s001.zip › File S1_Standard Operating Procedure (French version).pdf]

Supplementary File S1 - Standard Operating Procedure (French version)

Indications et prérequis

□ Confirme l'indication à l'i-gel® : ACR
□ Aspiration fonctionnelle à portée de main

Préparation du matériel

□ i-gel® de taille appropriée
□ Lubrifiant non siliconé (type KY)
□ Moyen de fixation (lacet, scotch, fixe-tube)
□ Capteur EtCO2

Mise en place du dispositif

□ Lubrifie suffisamment l'i-gel (sauf partie antérieure)
□ Compressions thoraciques continues dès le début et durant toute la pose du dispositif
□ Vérifie l'absence de corps étranger dans la bouche avant l'insertion
□ Ouvre la bouche en effectuant une bascule de la tête et/ou une traction de la mandibule
□ Effectue l'insertion de l'i-gel sans forcer (se positionne de lui-même avec précision et naturellement par-dessus la structure laryngée)
□ Connecte le ballon auto-remplisseur avec le filtre suivi du capteur $EtCO_2$

Evaluation

□ Vérifie le bon placement du dispositif à l'aide de la capnographie, le corrige au besoin
□ S'assure du bon fonctionnement du système et de l'efficacité de la ventilation

Suite de soins

□ Effectue le MCE en continu sauf si ventilation inadéquate en l'absence de pauses
□ Optionnel : sécurise l'i-gel® à l'aide d'un moyen de fixation
□ Optionnel : pose une sonde gastrique de taille adaptée et s'assure de sa position

Adaptations période COVID :

- l'équipement de protection des intervenants doit être maximal : au minimum masque FFP2, gants et lunettes de protection
- le filtre est monté directement sur l'i-gel® lors de sa préparation
- le reste de la procédure est inchangé
