# Supplementary material for: Effect of Early Supraglottic Airway Device Insertion on Chest Compression Fraction during Simulated Out-of-Hospital Cardiac Arrest: Randomised Controlled Trial
Source: J Clin Med. 2021 Dec 31;11(1):217. doi: 10.3390/jcm11010217 (PMC8745715; doi:10.3390/jcm11010217)
Supplement: Supplementary file 1 [file jcm-11-00217-s001.zip › File S2_Standard Operating Procedure_(English version).pdf]

Supplementary File S2 - Standard Operating Procedure (English version)

Indications and prerequisites

□ Confirms the i-gel® indication: cardiac arrest

□ Functional suction at hand

Preparation of material

□ Appropriately sized i-gel®

□ Non-silicone lubricant (type K-Y)

□ Means of attachment (lace, tape, tube holder)

□ EtCO2 sensor

Device insertion

□ Sufficiently lubricates the i-gel® (except the front part)

□ Continuous chest compressions from the start and during all device insertion

□ Check for the absence of foreign bodies in the mouth before insertion

□ Open the mouth by tilting the head and / or pulling the mandible

□ Performs the insertion of the i-gel® without forcing (positions itself precisely and naturally over the laryngeal structure)

□ Connect the resuscitator with the filter and the EtCO2 sensor

Assessment

□ Checks the correct placement of the device using capnography, correct it if necessary

□ Ensures the proper functioning of the system and the efficiency of the ventilation

Care suite

□ Performs continuous MCE unless ventilation is inadequate in the absence of interruptions

□ Optional: secure the i-gel® using a means of attachment

□ Optional: place a gastric tube of the appropriate size and ensure its position

COVID period adaptations:

- the protective equipment of the providers must be maximum: at least N95 mask, gloves and eyes protection
- the filter is placed directly on the i-gel® during its preparation
- the rest of the procedure is unchanged
