# Supplementary figures and images for: Effect of Early Supraglottic Airway Device Insertion on Chest Compression Fraction during Simulated Out-of-Hospital Cardiac Arrest: Randomised Controlled Trial
Source: J Clin Med. 2021 Dec 31;11(1):217. doi: 10.3390/jcm11010217 (PMC8745715; doi:10.3390/jcm11010217)

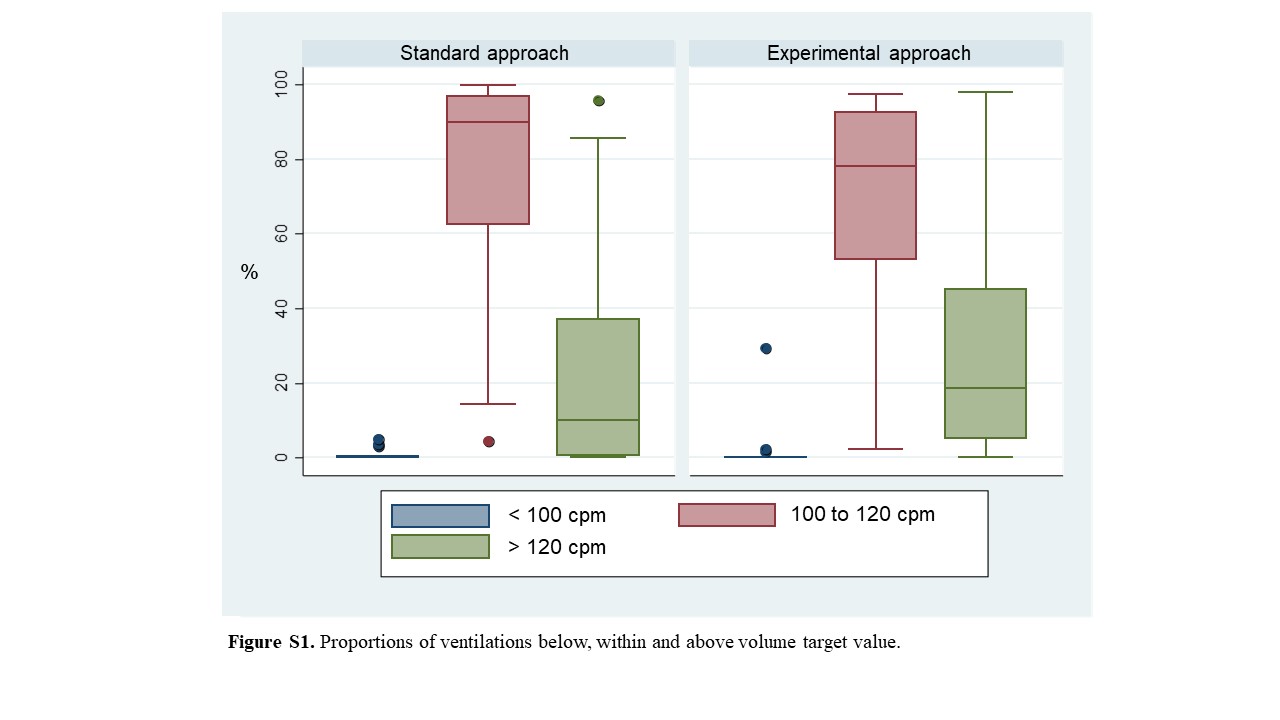

Supplement: Supplementary file 1 [file jcm-11-00217-s001.zip › Figure S1_Compression rate.jpg]

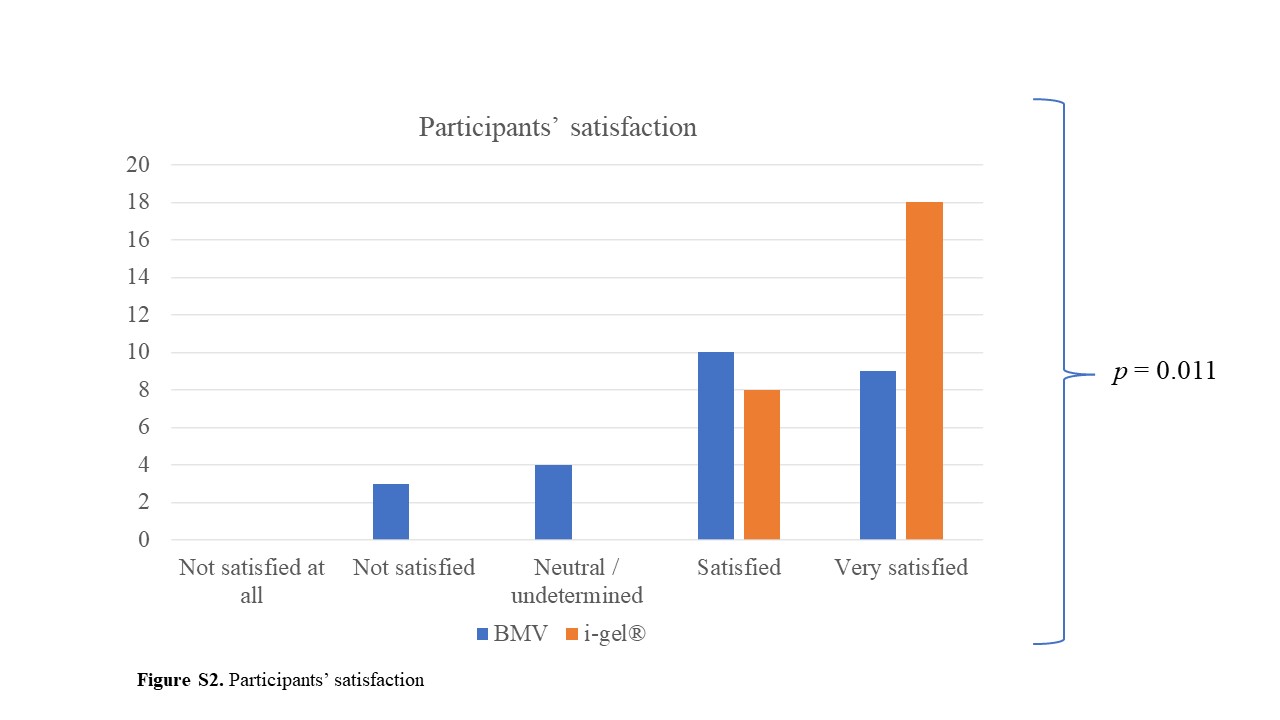

Supplement: Supplementary file 1 [file jcm-11-00217-s001.zip › Figure S2_Satisfaction.jpg]
